# Mechanistic studies of small molecule ligands selective to RNA single G bulges

**DOI:** 10.1093/nar/gkaf559

**Published:** 2025-06-25

**Authors:** Shalakha Hegde, Sana Akhter, Zhichao Tang, Chang Qi, Chenguang Yu, Anna Lewicka, Yu Liu, Kushal Koirala, Mikhail Reibarkh, Kevin P Battaile, Anne Cooper, Scott Lovell, Erik D Holmstrom, Xiao Wang, Joseph A Piccirilli, Qi Gao, Yinglong Miao, Jingxin Wang

**Affiliations:** Section of Genetic Medicine, Department of Medicine, Biological Sciences Division, University of Chicago, Chicago, IL 60637, United States; Department of Pharmacology and Computational Medicine Program, University of North Carolina, Chapel Hill, NC 27599, United States; Section of Genetic Medicine, Department of Medicine, Biological Sciences Division, University of Chicago, Chicago, IL 60637, United States; Analytical Research & Development, Merck & Co., Inc, Rahway, NJ 07065, United States; Calibr–Skaggs Institute for Innovative Medicines, The Scripps Research Institute, La Jolla, CA 92037, United States; Department of Biochemistry and Molecular Biology, Biological Sciences Division, University of Chicago, Chicago, IL 60637, United States; Department of Chemistry, Rockhurst University, Kansas City, MO 64110, United States; Department of Pharmacology and Computational Medicine Program, University of North Carolina, Chapel Hill, NC 27599, United States; Analytical Research & Development, Merck & Co., Inc, Rahway, NJ 07065, United States; New York Structural Biology Center, Upton, NY 11973, United States; Protein Structure and X-ray Crystallography Laboratory, University of Kansas, Lawrence, KS 66047, United States; Protein Structure and X-ray Crystallography Laboratory, University of Kansas, Lawrence, KS 66047, United States; Department of Molecular Biosciences, University of Kansas, Lawrence, KS 66045, United States; Analytical Research & Development, Merck & Co., Inc, Rahway, NJ 07065, United States; Department of Biochemistry and Molecular Biology, Biological Sciences Division, University of Chicago, Chicago, IL 60637, United States; Department of Chemistry, Physical Sciences Division, University of Chicago, Chicago, IL 60637, United States; Analytical Research & Development, Merck & Co., Inc, Rahway, NJ 07065, United States; Department of Pharmacology and Computational Medicine Program, University of North Carolina, Chapel Hill, NC 27599, United States; Section of Genetic Medicine, Department of Medicine, Biological Sciences Division, University of Chicago, Chicago, IL 60637, United States

## Abstract

Small-molecule RNA binders have emerged as an important pharmacological modality. A profound understanding of the ligand selectivity, binding mode, and influential factors governing ligand engagement with RNA targets is the foundation for rational ligand design. Here, we report a novel class of coumarin derivatives exhibiting selective binding affinity towards single G RNA bulges. Harnessing the computational power of all-atom Gaussian accelerated molecular dynamics simulations, we unveiled a rare minor groove binding mode of the ligand with a key interaction between the coumarin moiety and the G bulge. This predicted binding mode is consistent with results obtained from structure-activity relationship studies and transverse relaxation measurements by nuclear magnetic resonance spectroscopy. We further generated 444 molecular descriptors from 69 coumarin derivatives and identified key contributors to the binding events, such as charge state and planarity, by lasso (least absolute shrinkage and selection operator) regression. Our work deepened the understanding of RNA-small molecule interactions and integrated a new framework for the rational design of selective small-molecule RNA binders.

## Graphical Abstract

**Graphical Abstract:**
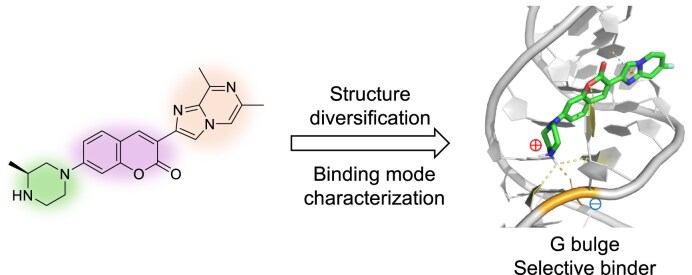

## Introduction

RNA plays critical roles in gene regulation and various cellular processes in almost all life forms, including transcription, translation, splicing, and epigenetic modifications [1, 2]. Selective targeting of RNA structures using small molecules is an important pharmacological modality that complements traditional protein targeting approaches [3–9]. For example, bacteria ribosomal RNA (rRNA) is an important antibiotic target with numerous clinically validated drug classes, such as aminoglycoside, tetracycline, macrolide, lincosamide, and oxazolidinone [10]. Recently, two synthetic compounds, risdiplam and branaplam, both targeting precursor messenger RNA (pre-mRNA)-U1 small nuclear ribonucleoprotein (snRNP) complex, attracted tremendous attention as RNA splicing modulators to treat genetic diseases, including spinal muscular atrophy [11–17] and Huntington’s disease [18–20]. We previously demonstrated that a class of coumarin analogues of risdiplam can induce GA-rich sequences to form loop-like structures using molecular dynamics (MD) simulations and proposed that this interaction in cells provided additional selectivity of the coumarin derivatives to the GA-rich SMN2 gene [21].

**Figure 1.**
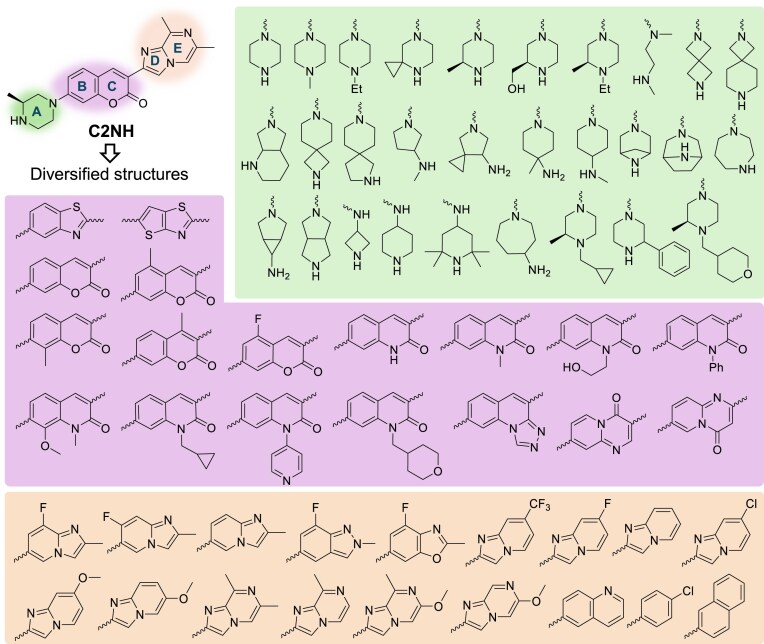
Molecular diversity of coumarin derivative analogues designed to bind bulged G RNA. The A, BC, and DE rings were replaced by the shaded green, purple, and orange structures, respectively (wavy lines = connecting bonds).

In addition to rRNA in bacteria and pre-mRNA in humans, several other classes of RNA have been targeted by chemical probes and drug candidates, including bacteria riboswitches [22–24], yeast self-splicing introns [25], microRNAs [26–31], untranslated regions (UTR) of mRNAs [32–34], and long non-coding RNAs [35–37]. In viruses, highly structured RNA regions have also been explored as targets for small molecules [38], such as an internal ribosome entry site in the 5′ UTR of the hepatitis C virus [39–42] and a transactivation response hairpin in human immunodeficiency virus 1 [43–45]. After the outbreak of SARS-CoV-2, we and others illustrated that the structural elements in the SARS-CoV-2 genome can also be targeted to suppress virus replication [46–51]. Specifically, we discovered that some coumarin derivatives (Fig. 1) can be repurposed to selectively bind to a single G bulge in 5′ UTR of SARS-CoV-2 without retaining splicing modulatory activities or binding to GA-rich loops [47]. We further demonstrated that covalently linking a ribonuclease (RNase) L recruiter and the coumarin-based G bulge binder yielded an active ribonuclease targeting chimera, which is effective in targeting SARS-CoV-2-infected epithelial cells [47].

In general, attaining selective and effective targeting of RNA using small molecules is a challenging endeavour due to several factors, including its conformational flexibility and heterogeneity and its polyanionic backbone, which all prevents the formation of deep hydrophobic binding pockets for small-molecule binding [8, 52]. Cheminformatics works have uncovered key factors governing the activity and selectivity of RNA binders [53–56] and have recently been further advanced by machine learning approaches [57]. However, the lack of methods for mechanistic studies of flexible RNA–small molecule ligand interactions critically limits further optimization of RNA ligands. A powerful approach to studying RNA-small molecule interactions is to use molecular dynamics (MD) simulations [21, 58, 59], which are able to fully account for the RNA flexibility on an atomistic level. Here, we present an integrated framework that combines all-atom Gaussian accelerated MD (GaMD) simulations, which can rapidly predict ligand binding modes, with nuclear magnetic resonance (NMR) transverse relaxation (*R*_2_) measurements and structure-activity relationship (SAR) studies, which can experimentally probe RNA–ligand interactions [21, 60]. We envision that our mechanistic studies of RNA ligands could serve as a model for understanding interactions between RNA and small molecules and for enhancing binding affinity across various RNA targets.

## Materials and methods

### Fluorescence polarization assay

Synthetic RNA oligonucleotides were procured from GenScript (Piscataway, NJ, USA) and reconstituted in nuclease-free water. Compounds were prepared at a concentration of 50 μM in dimethyl sulfoxide (DMSO) and were diluted in 2× assay buffer (50 mM 2-(*N*-morpholino)ethanesulfonic acid [MES], 100 mM NaCl, 0.004% TritonX, pH 6.1) to a concentration of 0.2 μM. A 1:3 dilution series of six points was prepared for each RNA in 20 μl water, resulting in concentrations ranging from 0.1 to 20 μM. Subsequently, 20 μl of 2× working solution containing the assay buffer and small-molecule ligand was added to each RNA sample in a 1:1 (v/v) ratio and mixed by pipetting. To measure fluorescence polarization (FP), 8 μl of the sample solution was transferred into a 384-well, black, flat-bottom microplate (Greiner, #784076) in triplicate. The plate was equilibrated at room temperature for 5 min prior to data acquisition using BioTek Synergy H1 (Winooski, VT, USA) with a blue FP filter cube (excitation/emission = 360/460 nm; BioTek, #8040563) at 25°C. Experimental data were analysed using the Prism 8.0 software package (GraphPad, San Diego, USA). A nonlinear curve fitting was employed to calculate the dissociation constant (*K*_d_), reported with a 95% confidence interval.

### Lasso regression

The structure of each of the 69 compounds was individually optimized using density-functional theory (DFT) calculation performed with Gaussian 09 software, employing the B3LYP functional and 6-31G(d) basis set in the ground state with default convergence criteria (see Supplementary data). The protonation state was predicted by Molecular Operating Environment (MOE) 2022 software (Chemical Computing Group, Montreal, Canada) at pH 7.0. The structures of all compounds were loaded on MOE 2022, and 443 molecular descriptors were generated using MOE 2022. Two molecular descriptors, h_pKa and h_pKb, were excluded because of the differential protonation states within the compound library. The dihedrals in degree units (°) between BC and DE rings within the DFT-optimized 3-dimensional (3D) structures were added as a new molecular descriptor. In the lasso regression analysis, the natural logarithm of the dissociation constant [Ln(*K*_d_), *K*_d_ in molar unit] was utilized as the dependent variable (*y*-value) (for the molecular descriptor values used for lasso regression, see MolecularDescriptors.csv). Lasso regression (L1 regularization) was performed in R (4.2.2) according to the reported protocol [61] (see LassoRegression.rmd for the R code). The non-zero coefficients were determined as described in Supplementary Table S1.

### GaMD simulations

GaMD is a computational enhanced sampling technique in which a harmonic boost potential is added to smooth the potential energy surface and reduce the system energy barriers [60]. GaMD does not require predefined collective variables or reaction coordinates and is thus advantageous for unconstrained enhanced sampling of complex biological systems such as ligand binding to highly flexible RNA. Since the GaMD boost potential exhibits a Gaussian distribution, the original biomolecular free energy profiles can be properly recovered through cumulant expansion to the second order [60]. GaMD has been successfully demonstrated on biomolecular simulations and revealed physical pathways and mechanisms of protein folding and ligand binding, which were consistent with experiments and long-timescale conventional MD simulations [62–66]. Recently, it was also applied to successfully capture ligand binding to single-stranded RNA [21]. Therefore, we adopt GaMD for RNA-ligand binding simulations in this study.

The RNA composer (available at https://rnacomposer.cs.put.poznan.pl) [67] was applied to build the simulation structure of target hairpin RNA with a G 1 × 0 bulge (RNA5: 5′-AAGAUGGAGAGCGAAACACACUCGUCUAUCUU). 3-Dimensional (3D) structures of three coumarin derivatives (C30, C30-Me, and SMSM64) were prepared using ChemBio Tool. All small molecules were protonated with a + 1 charge at the nitrogen in the piperazine ring. Parameterization of the small molecules was performed using antechamber with atomic charges calculated using the AM1-BCC option [68]. Each small molecule was initially placed at a distance of >15 Å away from the nucleic acid surface. Each simulation system was then prepared using the *tleap* module in AMBER 22 [69]. The system charges were neutralized in 0.15 M NaCl [70] and 0.01 M MgCl_2_ [71] solution. The AMBER OL3 force field [72, 73] was employed for RNA, the latest GAFF2 [74] for small molecules, and TIP3P [75] for water in the system. The AMBER OL3 force field was chosen for RNA because it has been optimized with specific parameters to model the intrinsic structural features of RNA, such as base pairing, stacking interactions, and high flexibility. These features are critical for accurately predicting how small molecule ligands interact with RNA. AMBER OL3 excels in capturing the delicate balance of interactions between RNA and ligands. The usage of AMBER OL3 and GAFF2 force fields has already been demonstrated to successfully capture ligand binding to single-stranded RNA [21] and is thus adopted in this study.

GaMD simulations were conducted using the *pmemd.cuda*, GPU-accelerating program in AMBER22 [69]. In all simulations, the hydrogen-heavy atom bonds were constrained using the SHAKE [76] algorithm, and the simulation time step was set to 2.0 fs. The particle mesh Ewald (PME) [77] method was employed to compute the long-range electrostatic interactions, and a cutoff value of 9.0 Å was applied to treat the non-bonded atomic interactions. The temperature was controlled using the Langevin thermostat with a collision frequency of 1.0 ps^–1^. Each simulation system was energy minimized using the steepest descent algorithm. This was followed by subjecting the system to heating from 0 K to 300 K for 200 ps. The system was further equilibrated using the constant number, volume, and temperature run for 800 ps at 300 K and constant number, pressure, and temperature ensemble at 300 K and 1 bar for 1 ns with 1 kcal/mol/Å^2^ constraints on the heavy atoms of the RNA and ligand. A short conventional MD simulation for 10 ns without any constraint was performed to collect initial potential statistics, including the maximum, minimum, average, and standard deviation (SD).

Dual-boost GaMD simulations were performed on binding of the three coumarin derivatives to target RNA. One boost potential was applied to the dihedral energetic term and the other to the total potential energetic term. The reference energy *E* for applying the boost potentials was set to the lower bound, i.e. *E* = *V*_max_. The average and SD of the system potential energies were calculated every 300 000 simulation steps (600 ps). The upper limit of the boost potential SD, σ0, was set to 6.0 kcal/mol for both the dihedral and the total potential energetic terms. The GaMD equilibration was carried out for 63 ns after adding the boost potential. Finally, three independent 1500 ns GaMD production simulations were conducted for each system with randomized initial atomic velocities.

### Simulation analysis

VMD [78] and CPPTRAJ [79] were used to perform trajectory analysis. The hierarchical agglomerative clustering algorithm [80] available in CPPTRAJ was used to conduct clustering of the ligand snapshots to identify low-energy conformations. The frames were sieved at a stride of 500 for clustering. The top 10 structural clusters were analysed to identify the representative conformations of each system. The center-of-mass distances were calculated between heavy atoms of the BC ring of the ligand and the RNA nucleotide G24 and between heavy atoms of the DE ring of the ligand and RNA nucleotide C12 using CPPTRAJ [79]. Furthermore, these distances were used as reaction coordinates for calculating a 2D potential of mean force free energy profile by reweighting all three GaMD simulations combined. The PyReweighting toolkit was used for reweighting the GaMD simulations [81]. A bin size of 1 Å was used for the ligand distances and the cutoff was set to 500 frames in a bin or cluster for reweighting.

### NMR experiments

An 84.1 nmol unlabelled RNA5 sample (Sigma–Aldrich) was dissolved in 135 μl of potassium buffer (25 mM potassium phosphate buffer, 50 mM potassium chloride, pH 6.2, 10% D_2_O) to prepare an ∼600 μM NMR sample. A similar sample condition was used in a previous report [82], where NMR assignment was determined for a ^15^N-labeled RNA containing the segment of RNA5.

All NMR spectra were acquired using a Bruker 800 MHz Ascend spectrometer equipped with a TCI cryoprobe at 298 K (Supplementary Fig. S1). RNA proton peak assignment was performed by comparing the measured ^1^H chemical shifts with literature values (Supplementary Table S2) [82]. Proton peaks were assigned if the chemical shift difference was <0.15 ppm. All NH protons of U and G residues that showed peaks in the 10−14 ppm region were assigned accordingly, except for the one from G24, where the literature assignment was missing. Two proton peaks observed in this chemical shift region that were not previously assigned should correspond to the NH of G24 as well as that of G13. G13 is a part of the linker that differs from the RNA sequence used in the literature. The proton peaks of G7-NH and U25-NH did not appear until the addition of a 70 μM ligand. All the unambiguously assigned resonances are summarized in Supplementary Table S2.

The ligand stock solution prepared for the titration experiment contained 10 mM C30 in DMSO-d6. 0.47, 0.47, 0.94, 1.88, 0.94, 1.88, and 2.82 μl of the stock solution were titrated into the RNA NMR sample to achieve final ligand concentrations of 35, 70, 140, 280, 350, 490, and 700 μM, respectively. Proton transverse relaxation rate *R*_2_ was measured from SOFAIR (band-selective optimized flip-angle internally encoded relaxation) (Supplementary Fig. S2) [83]. The band-selective excitation pulse p39 was centred at 12.1 ppm with a bandwidth of 5.2 ppm for NH region, and was centred at 7.6 ppm with a bandwidth of 3 ppm for NH_2_/aromatic region. Transverse relaxation was encoded through the incrementation of a delay *t* flanking the refocusing pulse p40. The delay time was set to between 0 and 0.4 s with a total of 12 increments (0, 0.002, 0.005, 0.010, 0.015, 0.020, 0.025, 0.030, 0.050, 0.1, 0.2, and 0.4 s). The duration of each experiment is ∼18.5 min. Data were processed and analysed using MestReNova. *R*_2_ of each resonance was determined through area integration and fitting the integrals to the following equation: $I = {{I}_0}{{e}^{ - t{{R}_{\rm 2}}}} + B$, where *t* is the delay time, *R*_2_ is the transverse relaxation rate, and *B* is a constant to account for any baseline differences between experiments.

## Results

### Coumarin derivatives selectively bind to RNA G (1 × 0) bulge

The prototype coumarin derivative C2NH, which binds to RNA single G bulges (denoted as G 1 × 0 bulge) at a moderate binding affinity, contains five heterocyclic rings: piperazine (A), coumarin (BC), and a [5,6]-fused ring (DE) (Fig. 1). We previously reported C2NH as an active splicing modulator that can bind to a GA-rich loop within the SMN2 gene [21]. We modified the E ring to remove the splicing modulatory activity and repurposed the scaffold to other RNA targets, resulting in a potent G 1 × 0 bulge binder that strongly associates with a structural motif in the RNA genome of SARS-CoV-2 [21, 47]. To further probe the mechanism of the coumarin derivative in RNA binding interaction, we synthesized a collection of 69 analogues of C2NH (Fig. 1). Each compound in this collection comprises at least one ring distinct from the parent compound. For instance, in Ring A, the piperazine was replaced by cyclic amines of varying sizes. In Ring BC, the coumarin was substituted by other heterocycles with various substituents. Similarly, the [5,6]-fused Ring DE was replaced by [6,5]- or [6,6]-fused rings (Fig. 1).

**Figure 2.**
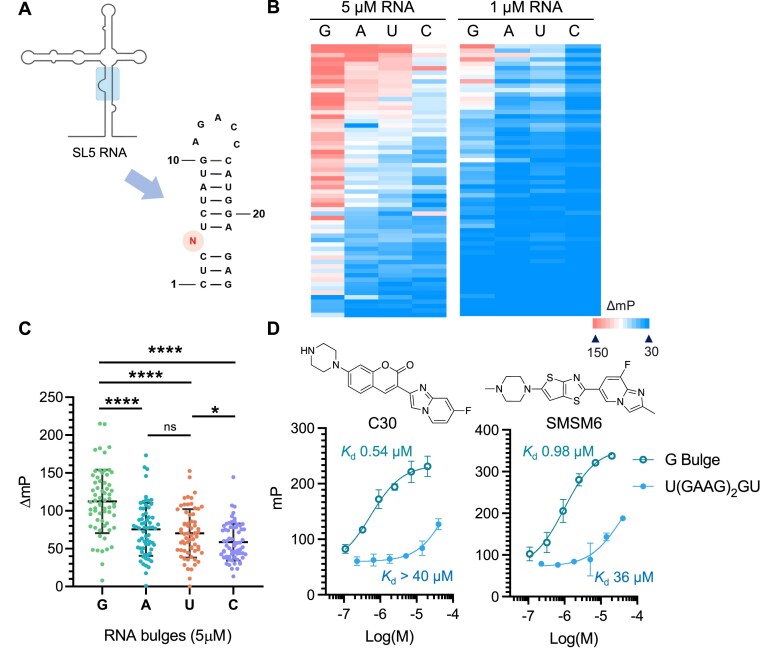
Coumarin derivatives can selectively bind to RNA G 1 × 0 bulges: (**A**) RNA structures of the 1 × 0 RNA bulges used for *in vitro* binding profiling using the FP assay. *N* = *G*, *A*, *U*, or *C* (RNA1-4). (**B**) Heatmap profile of the ΔmP = (FP^RNA-ligand^ – FP^ligand^) × 1000 for RNA binders in the presence of [RNA] = 5 or 1 μM (red = high polarization, blue = low polarization). (**C**) ΔmP of RNA-ligand complex for RNA ligands at 5 μM. Each data point represents a measurement of a ligand in the 69-compound collection. **** indicates *P* < .0001. (**D**) Dose-response curves for compounds (SMSM6, C30) selectively binding to the bulged G RNA (RNA1) compared to an 11-nucleotide GA-rich sequence that would form a double loop-like RNA structure measured by the FP assay.

All compounds in this collection are fluorescent with an excitation/emission wavelength at ∼400/480 nm, which allowed us to use FP assay to rapidly determine their binding affinity to the bulge G RNA. Using a bulged G RNA segment from SARS-CoV-2 SL5 RNA as a model, we extensively profiled this 69-compound library against all four 1 × 0 RNA bulge variants (RNA1–4) for binding affinities (Fig. 2A, Supplementary Figs S3 and S4, and Supplementary Table S3). Almost all binding molecules showed superior selectivity for the G bulge compared to other RNA bulges (bulged A, U, and C), as judged by the polarization change (ΔmP) at two concentrations (1 and 5 μM) (Fig. 2B).

Statistical analysis of all 69 compounds revealed that the binding affinity for different RNA bulges followed the following trend: G >> A ≈ U > C (Fig. 2C). Since C2NH can also bind to GA-rich RNA loops [21] via an induced-fit mechanism, resulting in the formation of a double loop-like structure, we also tested the binding of coumarin derivatives to a flexible GA-rich RNA (5′-U(GAAG)_2_GU) (Supplementary Fig. S5 and Supplementary Table S4). Interestingly, certain compounds, such as C30 and SMSM6, demonstrated >35-fold selectivity towards bulged G over GA-rich RNA (Fig. 2D), while a few compounds bind to both RNAs with comparable binding affinities (Supplementary Fig. S6). This suggests that a portion of coumarin derivatives may employ a distinct binding mechanism to target the RNA 1 × 0 G bulge selectively. The binding affinity of selected G bulge binders to RNA1 was validated using the microscale thermophoresis assay, which provided consistent results. (Supplementary Fig. S7).

### GaMD simulations captured spontaneous binding of coumarin derivatives to RNA G (1 × 0) bulges in the minor groove

To explore the binding of specific RNA G bulge ligands, we performed all-atom simulations using the GaMD method [60] on binding of three coumarin derivatives to the model hairpin RNA5 with a G bulge (Supplementary Fig. S8A; see simulation details in “Materials and methods” section).

**Figure 3.**
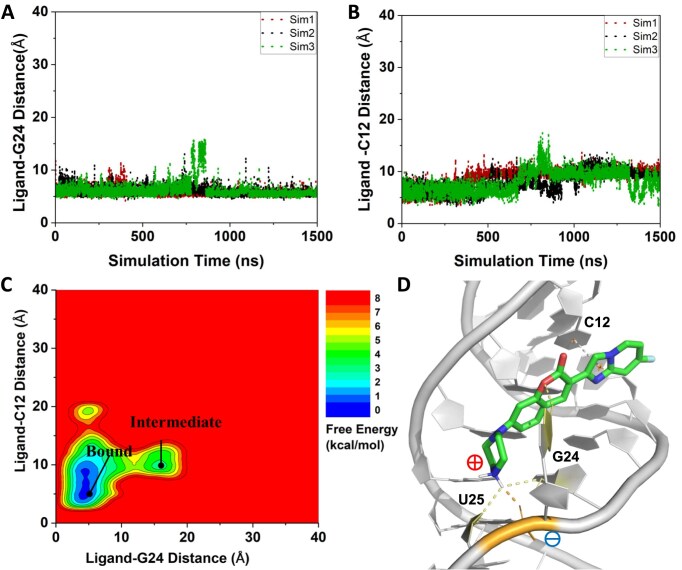
GaMD simulations captured stable binding of coumarin derivative C30 to RNA5: (**A**) Time courses of the center-of-mass distance between heavy atoms of the BC ring of ligand C30 and the RNA bulge G24 were calculated from three 1500 ns GaMD production simulations. (**B**) The center-of-mass distance between heavy atoms of the DE ring of ligand C30 and RNA nucleotide C12 is plotted as a function of simulation time. (**C**) 2D free energy profile calculated with all three GaMD simulations combined, showing two distinct low-energy states, namely the ‘Bound’ and ‘Intermediate’. (**D**) Representative conformation of RNA−C30 complex in the bound state (grey dash line = π–π stacking, yellow dash line = hydrogen bonding, orange dash line = ionic interaction). The ‘Intermediate’ conformation is shown in Supplementary Fig. S9.

We found that C30 bound spontaneously to the G bulge and minor groove of RNA5 during the GaMD equilibration simulation (Supplementary Fig. S8B). It then maintained the bound conformation in three independent 1500 ns GaMD production simulations (Fig. 3). C30 remained bound to the RNA5 minor groove throughout nearly the entire three 1500 ns GaMD production simulations, despite small fluctuations around ∼800 ns in Sim3. Upon binding to the minor groove of RNA5, a short distance was observed between the coumarin core (BC ring) of the ligand and the bulged G24 at 3.5–5 Å (Fig. 3A). Moreover, a π–π stacking interaction was observed between C12 and the fused D/E ring of the C30 ligand in simulations (Fig. 3B). We used these distances as reaction coordinates to further calculate a 2D free energy profile of C30 binding to RNA5, which showed two low-energy states, designated as ‘Bound’ (more stable) and ‘Intermediate’ states (Fig. 3C; bound state structure was deposited in Model Archive Project ma-q6hl4). To experimentally probe the minor groove binding mechanism that we observed in the GaMD simulations, we conducted additional FP binding assays using C30 and various DNA versions of the RNA G bulge sequences (same sequences as RNA1 and RNA5). Our results show that the deoxyribose modification gives rise to a >13-fold decrease in binding affinity (Supplementary Fig. S10). This result differed from what we observed with GA-rich loop binders, where the DNA aptamers bind to the ligands with a higher binding affinity than the RNA aptamers with the same sequences [21]. Given that double-stranded (ds) DNA typically adopts a different groove geometry than dsRNA [84], the reduced binding of C30 to DNA supports a groove-binding mechanism and highlights the RNA selectivity in C30 ligand recognition.

In the ‘Bound’ state, C30 formed three primary interactions within the minor groove of RNA5 (Fig. 3D): (i) The bulged G (G24) contributed to a hydrogen bond via its N1 position to the coumarin lactone moiety in C30. (ii) A phosphate group in the RNA backbone was involved in an ionic interaction with the protonated NH_2_^+^ group in the piperazine ring of C30. (iii) Nucleotide C12 formed π–π stacking interactions with the ligand C30 in the RNA minor groove. In the transient ‘Intermediate’ state, C30 was located at a much larger distance from the G24 nucleotide and did not insert into the RNA minor groove (Supplementary Fig. S9).

**Figure 4.**
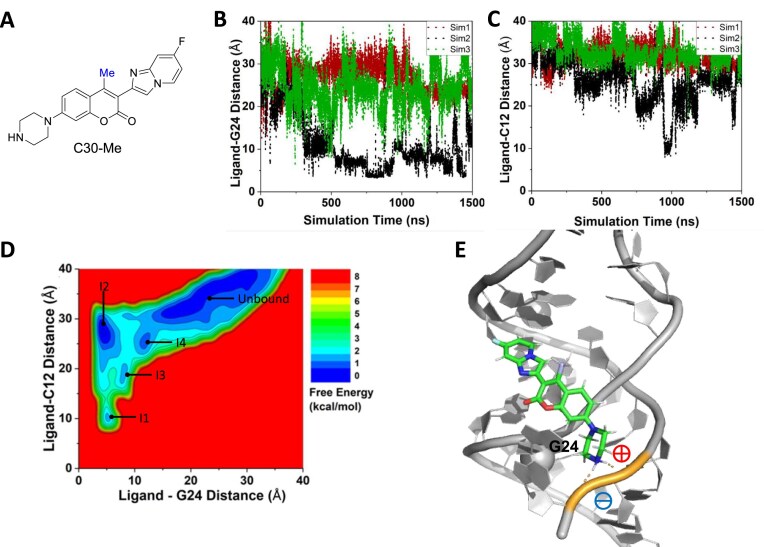
GaMD simulations captured transient binding of ligand C30-Me to RNA5: (**A**) Chemical structure of C30-Me. (**B**) Time courses of the center-of-mass distance between heavy atoms of the coumarin core (BC ring) in the ligand and the RNA bulged G24 calculated from three independent 1500 ns GaMD simulations. (**C**) The center-of-mass distance between the heavy atoms of the DE ring of C30-Me and RNA nucleotide C12 plotted as a function of simulation time. (**D**) 2D free energy profile calculated with all three GaMD simulations combined, showing five low-energy states, namely the ‘I1’, ‘I2’, ‘I3’, ‘I4’, and ‘Unbound’. (**E**) Representative conformation of C30-Me–RNA5 complex in the I1 state (orange dash line = ionic interaction).

We further performed GaMD simulations on two inactive analogues of C30, namely C30-Me (Fig. 4A) and SMSM64 (Supplementary Fig. S11). C30-Me merely has an additional methyl group on the C ring compared to C30, which would break the planarity of Rings BC and DE within the compound (see discussions below). On the other hand, SMSM64 has an N-pyridinyl quinolone replacing the coumarin core, whose bulkiness might block the polar interaction with the RNA G bulge. In experiments, both compounds exhibited >100-fold reduced binding affinities towards RNA5, with SMSM64 displaying a dissociation constant (*K*_d_) of >50 μM, in comparison to C30, which has a *K*_d_ of 0.27 ± 0.01 μM to RNA5 (Supplementary Fig. S12). Similar binding affinities were observed for these compounds when binding to RNA1 (see Supplementary Table S3).

In all three 1500 ns GaMD simulations for C30-Me, the ligand seldom reached the target site in the minor groove of RNA5 (Fig. 4). In the situation where C30-Me transiently interacted with G24 nucleotide (‘Sim2’ in Fig. 4B), the ligand remained out of the RNA minor groove with a distance >10 Å from nucleotide C12 (Fig. 4C). Altogether, four transient binding states were identified from the free energy profile, designated as ‘Intermediate’ states I1–I4, as well as the ‘Unbound’ state, where the ligand dissociated from the RNA (Fig. 4D and Supplementary Fig. S13). The presence of multiple intermediate states suggested that the ligand explored various binding positions but was unable to achieve stable insertion into the minor groove. These intermediate conformations all maintained ionic interactions between the positively charged piperazine ring on the ligand and at least one phosphate group on the RNA backbone (Fig. 4E). For SMSM64, the ligand remained mostly >∼15 Å away from key nucleotides G24 and C12 throughout the 1500 ns GaMD simulations (Supplementary Fig. S11B and C). The resulting free energy profile showed only an ‘Unbound’ state (Supplementary Fig. S11D and E).

During the GaMD simulations, we observed high fluctuations of G24 in RNA5 in the absence of stable ligand binding as for the C30-Me and SMSM64 coumarin derivatives. In comparison, stronger binding of C30 to RNA5 led to significantly reduced root-mean-square fluctuations of the RNA nucleotides, especially G24 (Supplementary Fig. S14). Experimentally, we screened ∼20 crystal structures of RNA1 obtained using fragment antigen-binding region (Fab) chaperon-assisted crystallography (for a representative structure, see Protein Data Bank with accession code 9DN4) and observed dynamic conformations of the G bulge nucleotide [85], whereas other nucleotides remained relatively static (Supplementary Fig. S15 and Supplementary Table S5). This result is also consistent with our chemical probing results in SARS-CoV-2 RNA, where a high SHAPE (selective 2′ hydroxyl acylation analysed by primer extension) signal was observed with high concentrations of acylation agents (e.g. 10 mM FAI-N3) [47].

### NMR validation of the minor groove binding mode

Next, we used NMR experiments to validate the predicted binding mode between coumarin analogues and the RNA with a G 1 × 0 bulge. First, we assigned imino protons and some other protons on the nucleobases in ^1^H NMR using a reported assignment that contains the segment of RNA5 [82]. The assigned peaks were distinguishable ones within 0.15 ppm from the reported ^1^H NMR chemical shifts (Supplementary Table S2). Next, we applied a recently published NMR method, ^1^H SOFAIR (band-Selective Optimized Flip-Angle Internally-encoded Relaxation) [86], to quantify *R*_2_ relaxation rate of the receptor signals in order to characterize RNA-ligand interactions. *R*_2_ relaxation reflects on dynamics and motion changes of molecules, which is sensitive to weak binding (*K*_d_ ∼μM), and has been widely utilized as an NMR approach for identifying the binding sites of biomolecules [87, 88]. Here, the SOFAIR pulse sequence [86] was utilized to facilitate signal acquisition with high sensitivity of an RNA sample at mM concentration. Notably, SOFAIR was specifically designed to speed up data acquisition, and in this instance, led to a reduction in acquisition time from several hours, characteristic of conventional proton *R*_2_ measurements using Carr-Purcell-Meiboom-Gill type of methods [89, 90] to ∼20 min.

**Figure 5.**
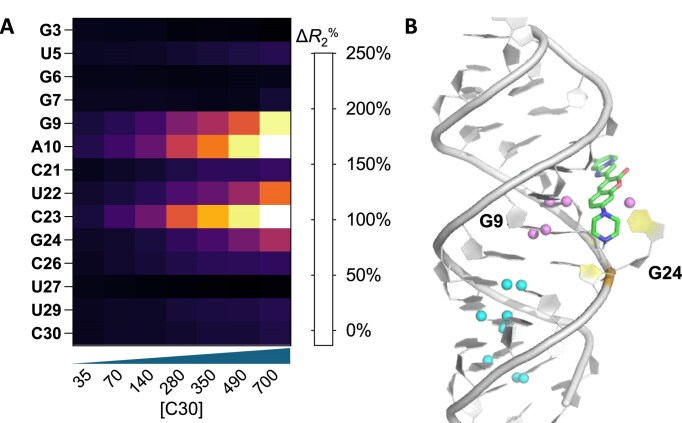
NMR relaxometry validation of the minor groove binding mode: (**A**) Normalized *R*_2_ relaxation rate percentage changes obtained from each assigned proton of RNA nucleotide in the absence and presence of the ligand C30. The coloured columns in the bar plot represented *R*_2_ measured at different ligand concentrations. Normalized *R*_2_ values were calculated using the equation: $\Delta R_2^\% = \ \frac{{R_2^{{\rm ligand} + } - R_2^{{\rm ligand} - }}}{{R_2^{{\rm ligand}{\rm } - }}} \times 100\%$. (**B**) Overlay of the simulation-predicted ‘Bound’ state of C30-RNA5 and all identifiable protons in ^1^H SOFAIR (pink = increased Δ*R*_2_^%^; blue = unchanged or decreased Δ*R*_2_^%^).

The *R*_2_ relaxation rates were obtained from RNA nucleobases during the titration of the ligand C30 (Supplementary Table S6). As shown in Fig. 5A, the titration of C30 induced an overall *R*_2_ change, indicating binding between C30 and the bulged G RNA. The most pronounced increase in *R*_2_ was observed in G9, A10, U22, C23, and G24, implying the direct involvement of these nucleotides in binding. In contrast, the relaxation rates observed from G3 to G7 and C26 to C30 exhibited much smaller increases or even negative changes upon ligand addition, suggesting that these regions of the RNA are not directly involved in binding. These findings from NMR experiments regarding the bound and unbound RNA nucleotides are consistent with those obtained from the GaMD simulations (Fig. 5B). Interestingly, the putative binding location is selective to one side of the G bulge (U22–G24), implying sequence selectivity in the minor groove. It is worth noting that only a few small molecular ligands have been reported as minor groove binders (e.g. PDB 1QD3) [91], likely because the minor groove is wide and shallow in A-form dsRNAs. In summary, our NMR data strongly supported a minor groove binding mechanism for C30, as the ligand is unlikely to bind to the major groove of the RNA given the observed *R*_2_ relaxation changes. This finding further highlights the critical role of the bulged G in ligand interactions within this unusual minor groove binding mechanism.

### Molecular features on the ligands for RNA binding

We also determined how the molecular characteristics of these ligands contribute to their efficacy in RNA G bulge binding. Our approach involved an SAR analysis using regularized regression based on *in vitro* binding affinity data. Given the similarities in shape, size, and molecular scaffolds of our 69-compound library, we expected the SAR analysis to offer detailed molecular insight into the specific structural and electronic properties responsible for their potency.

We used MOE software to individually predict the most likely protonation state based on the 2D structures of each molecule. The majority of molecules were found to be mono-protonated at the aliphatic cyclic amine (A ring), with a few exceptions that carried two positive charges (Supplementary data). We then optimized the 3D structure of each molecule using *ab initio* DFT calculation with B3LYP 6–31G(d) basis set (Supplementary data). Using these 3D structures as input, we generated 443 molecular descriptors using MOE software (Supplementary data). To account for the planarity of the coumarin derivatives, we introduced a new dihedral descriptor between the aromatic rings BC and DE based on the most stable conformer predicted by DFT calculations. We then used the least absolute shrinkage and selection operator (Lasso) regression technique to identify the important electronic and structural features among these molecular descriptors using a modified analytical pipeline [57, 61]. Lasso regression is a linear regression approach used for feature selection, which effectively eliminates unimportant variables. This process resulted in 16 molecular descriptors that significantly contributed to the binding affinity (Supplementary Table S1), of which eight molecular features are related to the charge and shape of the RNA ligands (Table 1).

**Table 1.** Molecular descriptors selected by lasso regression for RNA binding^a^

Molecular descriptor	Description^a^	Class/impact^b^
FCharge	Total formal charge of the molecule.	Charge/+
a_base	Number of basic atoms.	Charge/+
PEOE_VSA_FPOS	Fractional positive VDWSA.	Charge/+
PEOE_VSA_FNEG^c^	Fractional negative VDWSA.	Charge/−
PEOE_VSA_NEG	Total negative VDWSA.	Charge/−
NPR1	PMI^d^ ratio: PMI1/PMI3	Shape/−
std_dim1	The square root of the largest eigenvalue of the covariance matrix of atomic coordinates.	Shape/+
*ω*(BC–DE)	Dihedral between BC and DE rings in the optimal structure.	Shape/–

^a^VDWSA = van der Waals surface area.

^b^+ and – signs indicate variables positively or negatively correlated to the binding affinity. ^c^PEOE_VSA_FNEG = –PEOE_VSA_FPOS. ^d^PMI = Normalized principal moments of inertia.

The five charge-related molecular descriptors were based on the total formal charge of the molecule (FCharge), the number of basic atoms that can be potentially protonated in physiological pH (A_base), the fractional positive (PEOE_VSA_FPOS) and negative (PEOE_VSA_FNEG) charges per unit area, and the total negative charge per unit area (PEOE_VSA_NEG). Since RNA is densely negatively charged, it is reasonable that positive charges would significantly contribute to RNA binding due to charge attraction. In the GaMD simulations with C30, intermolecular ionic interactions between the positive charge on the piperazine ring of C30 and phosphate groups on the RNA backbone were critical in maintaining the stability of the RNA-ligand complex. When we acetylated the piperazine ring of C30 at the N4 position (C30-Ac) to prevent protonation, the binding affinity decreased by a factor of >5, highlighting the importance of electrostatic interaction between the ligand and RNA (Supplementary Fig. S16). We further hypothesized that the ligand used the positive charge on Ring A to explore suitable binding pockets at the early stage of the binding process. This hypothesis was supported by GaMD simulations, in which we observed all identifiable transient binding states (‘Intermediate’ states) of C30–RNA5 and C30-Me–RNA5 complexes retained an ionic interaction with RNA backbone phosphates (Supplementary Fig. S9 and S13; Supplementary Movie S1).

**Figure 6.**
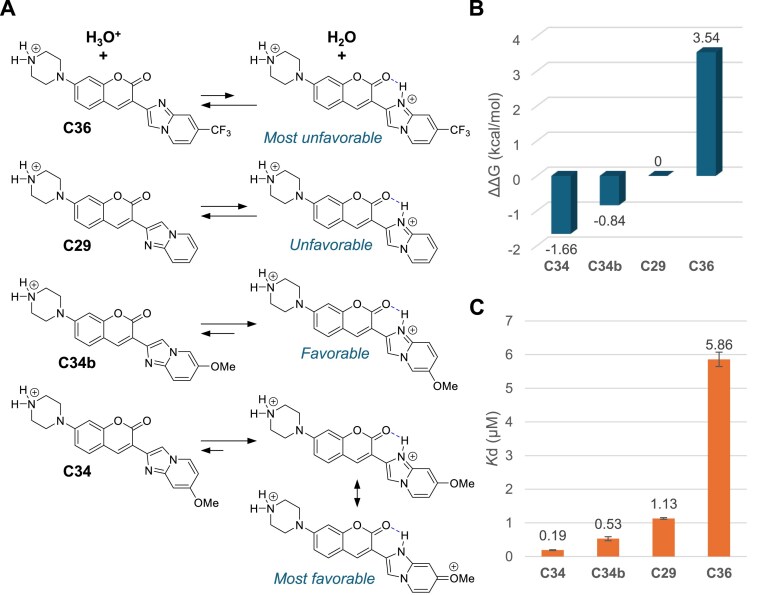
Protonation state of the ligands contributes to the RNA binding. (**A**) Equilibria for the protonation reactions of four coumarin derivatives. (**B**) Protonation energy (relative to C29) was calculated using DFT with B3LYP 6–31G(d) basis set. (**C**) Observed binding affinity of the four compounds.

We also verified the impact of local positive charges on coumarin derivatives on *in vitro* binding by selecting four compounds, C29, C36, C34, and C34b, which only differ in the structures of the E ring. These compounds have two potential protonation sites: a piperazine A ring and an imidazole D ring. The second protonation site on the D ring can be partially stabilized by the coumarin moiety by forming an internal hydrogen bond. We speculated that the propensity of imidazolium formation significantly depends on the substituents on the E ring (Fig. 6A). For example, substituting the E ring with a trifluoromethyl group makes the molecule less amenable to protonation due to the electron-withdrawing effects. In contrast, the presence of an electron-donating methoxy group in compound C34b enhances the favourability of imidazolium formation. When the methoxy group is positioned at the 4′ location (C34), the existence of a resonance structure further contributes to stabilizing the positive charge (Fig. 6A). We verified the protonation energy of the four compounds relative to C29 using DFT calculations and compared it with the *in vitro* binding data (Fig. 6B and C). The dissociation constants for these four compounds exhibit a consistent trend concerning protonation energy, providing compelling evidence that local positive charges on ligands significantly contribute to RNA binding.

The 3D shape descriptors also strongly correlate with the binding affinity (Table 1). For example, NPR1 and NPR2 are numeric shape descriptors with values between 0 and 1 that characterize the general 3D geometries of molecules [92]. All compounds in our compound collection exhibit a small NPR1 value (<0.2) and a large NPR2 value (>0.85), indicating rod-like molecular structures. This observation is consistent with a prior cheminformatic analysis of diverse RNA-binding molecules [54]. In addition, the positive contribution of the shape descriptor std_dim1 indicates that a longer molecule makes the ligand more favorable for binding, which is consistent with our expectations for groove binders.

**Figure 7.**
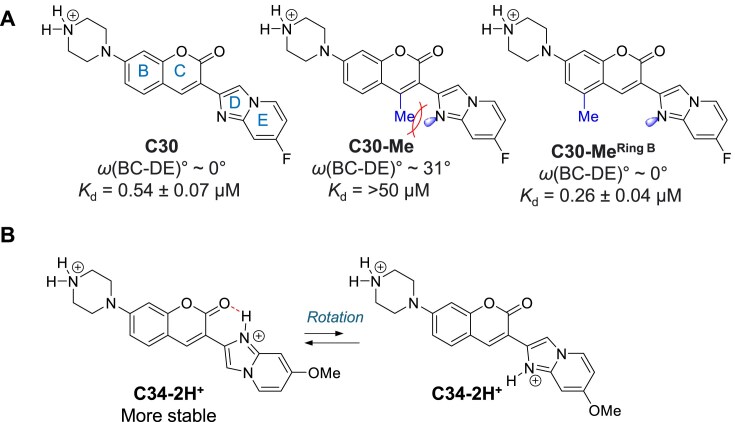
Planarity of the ligands contributes to the RNA binding. (**A**) The dihedral angle of Rings BC and DE [*ω*(BC–DE)] of the ligands in their ground states demonstrates a positive correlation with the dissociation constant (*K*_d_). Methyl group on the Ring C can introduce steric clashes between Rings BC and DE, reducing their co-planarity. (**B**) Di-protonated C34 maintains planarity in both conformations due to the flipping of the DE ring. The left conformation is stabilized by internal hydrogen bonding at the imidazolium ring.

Finally, we observed a positive correlation between planarity and binding affinity, as indicated by the inverse relationship between the dihedral angle of Rings BC and DE [*ω*(BC–DE)] and the natural logarithm of the binding constant (Ln*K*_d_). In C30, the dihedral angle between the BC–DE ring is ∼0, making it a planar molecule (Supplementary Fig. S17), which facilitates groove binding. However, adding a methyl group on ring C of C30 (C30-Me) causes steric hindrance between the methyl group and the lone pair electron of the imidazole nitrogen, disrupting the planarity of the molecule, rending C30-Me a poor binder (Fig. 7A and Supplementary Fig. S17). We also tested the role of this methyl group on ring B (C30-Me^RingB^), where the methyl group no longer sterically clashes with the imidazole ring. As expected, C30-Me^RingB^ is planar in its most favourable conformation (Supplementary Fig. S17), and the binding affinity was comparable to that of C30 (Fig. 7A). Planarity might also contribute to the high binding affinity of C34 (*K*_d_ = 0.10 ± 0.01 μM to RNA1). In the second protonation site of C34, the imidazole ring can form an internal hydrogen bonding with the coumarin lactone, further stabilizing the planar conformation (Fig. 7B and Supplementary Fig. S17).

## Discussion

In this study, we have reported a new group of coumarin derivatives that exhibit selective binding to bulge G RNA. Using all-atom GaMD simulations, NMR, and SAR studies, we have identified critical interactions that permit minor groove binding as well as crucial molecular properties of the ligands that significantly contribute to their binding affinity to bulged G RNAs. The ligand-RNA minor groove binding interface was validated by ^1^H SOFAIR NMR experiments that can rapidly characterize the ligand binding behaviour on RNAs. Our research establishes a new example for understanding RNA-small molecule interactions with nanomolar binding affinity.

## Supplementary Material

gkaf559_Supplemental_Files

## Data Availability

The representative C30-RNA5 bound conformation generated by GaMD simulations is available in PDB format in the Model Archive repository (https://modelarchive.org) under project ma-q6hl4. The representative apo RNA crystal structure of RNA1 is available in PDB under accession number 9DN4. NMR data is available in BMRB repository (https://bmrb.io/): https://deposit.bmrb.io/entry/load/93501976-1149-4710-989d-f797df292649.
