# Supplementary material for: Mechanistic studies of small molecule ligands selective to RNA single G bulges
Source: Nucleic Acids Res. 2025 Jun 25;53(12):gkaf559. doi: 10.1093/nar/gkaf559 (PMC12188297; doi:10.1093/nar/gkaf559)
Supplement: gkaf559_Supplemental_Files [file gkaf559_supplemental_files.zip › Caption for the supplementary movie.docx]

Caption for the supplementary movie: "Simulated C30-RNA5 binding pathway".
